# Nutritional status and associated factors among adult tuberculosis patients in public health centres of Horro Guduru Wollega Zone, Oromia Region, Western Ethiopia

**DOI:** 10.1017/jns.2024.79

**Published:** 2024-11-26

**Authors:** Dessalegn Obsina, Abeza Mitiku Kera, Asrat Zewdie Zenebe, Sisay Teferi, Abonesh Taye, Tefera Belachew

**Affiliations:** 1 Jimma Gennete Woreda Health Office, Horro Guduru Wollega Zone, Ethiopia; 2 Department of Public Health, College of Health Science, Mattu University, Mattu, Ethiopia; 3 Department of Medical Laboratory Science, College of Health Science, Mattu University, Mattu, Ethiopia; 4 Department of Nutrition and Dietetics, Faculty of Public Health, Institute of Health, Jimma University, Jimma, Ethiopia

**Keywords:** Ethiopia, Horro Guduru Wollega, Tuberculosis, Under nutrition, BMI, Body Mass Index, DDS, Dietary Diversity Score, DOTS, Directly Observed Treatment Short course, FFQ, Food Frequency Questionnaires, HFIAS, Household Food Insecurity Assess Scale, TB, Tuberculosis

## Abstract

This study aimed to assess nutritional status and associated factors among adult tuberculosis patients in public health centres in Horro Guduru Wollega Zone, Western Ethiopia, 2021. An institutional-based cross-sectional study was conducted among 334 randomly selected adult TB patients at public health centres from May 7, 2021, to June 21, 2021. Data were collected using structured questionnaires and anthropometric measurements. The nutritional status was measured by using body mass index (BMI). Data was entered into EpiData version 4.6 and exported to SPSS version 25 for analysis. A bivariate and multivariable multinomial logistic regression analysis was done to identify factors associated with nutritional status. The prevalence of under and overnutrition was found to be 48.2% and 8.7%, respectively. Female TB patients (AOR = 3.48, 95% CI: (1.918–6.314)), patients who didn’t receive dietary counselling (AOR = 2.51, 95% CI: (1.335–4.720)), TB patients on the initiation phase of treatment (AOR = 3.76, 95% CI: (2.072–6.852)), and meal frequency less than three times per day (AOR = 3.6, 95% CI: (1.942–6.676)) were significantly associated with under nutrition. The prevalence of undernutrition was high in the study area. Being a female, being in the initiation phase of treatment, lack of dietary counselling, and having meal less than three per day were independently associated with undernutrition. Hence, regular nutritional assessments, dietary counselling, and nutritional support should be encouraged at the facility and community level.

## Introduction

Tuberculosis (TB) is a disease caused by bacteria (mycobacterium tuberculosis) that mostly affects the lungs and, infrequently, other organs.^(1)^ People infected with TB bacteria have latent TB and have a 5–15% lifetime chance of developing active TB.^(2)^ Individuals with impaired immune systems, such as those with HIV/TB co-infection, malnutrition, diabetes or tobacco use, are more likely to develop TB.^(3–5)^ Patients with TB are more likely to have a loss of appetite, poor nutrient absorption, and a slow metabolism.^(1,5)^

The risk of developing complications, including death, from infections is influenced by an individual’s nutritional status.^(6)^ However, nutritional status and nutrient utilisation are also negatively affected following infection.^(7)^ Patients with TB are at a high risk of losing their appetite, impaired nutrient absorption, and having lower metabolism, all of which can lead to body wasting.^(1,5)^

Malnutrition and TB have a bidirectional relationship; having active TB causes weight loss, and being underweight is a known risk factor for developing TB, whether through the reactivation of latent TB or the development of progressive primary disease following infection.^(6–9)^

Geographically, the majority of TB cases were within the WHO regions of South-East Asia (44%), Africa (24%), and the Western Pacific (18%), with smaller percentages within the Eastern Mediterranean (8%), North America (3%), and Europe (3%). Eight countries that accounted for two-thirds of the worldwide total were: India (27%), China (9%), Indonesia (8%), the Philippines (6%), Pakistan (6%), Nigeria (4%), Bangladesh (4%), and South Africa (3%).^(10)^

Despite the 42% decrease in the annual TB incidence from 369 cases per 100,000 populations in 1990 to 177 per 100,000 populations in 2016, Ethiopia remains to be among the 30 countries reported with a high burden of TB, TB/HIV, and drug-resistant tuberculosis (DR-TB) for 2015 to 2020.^(2,11)^

Malnutrition is a strong risk factor for progression from TB infection to disease and is estimated to contribute to 26 % of incidents of TB globally.^(12)^ Despite the high prevalence of malnutrition and mortality in African countries, including Ethiopia there is insufficient implementation of counselling and nutritional care for TB patients.^(10,13)^

According to studies conducted in various regions of Ethiopia the prevalence of undernutrition among adult TB patients that ranges from 39.7% in Addis Ababa^(14)^ to 63.2% in the Oromia region.^(15)^ Sex, low educational status, having an eating problem, marital status (being single or widowed), types of TB, HV/TB co-infection, duration of treatment, alcohol consumption, high family size, low family income, functional status, lack of dietary counselling, lack of nutrition care and support were all factors associated with under nutrition in Ethiopia.^(16–18)^ However, there were no studies that have linked dietary patterns to malnutrition in TB patients in Ethiopia. Dietary pattern is used as a tool to measure dietary quality, micronutrient adequacy, and food access. Inadequate dietary quality and diversity lead to undernutrition including, underweight, and wasting.^(19)^ Therefore, the purpose of this study was to determine the nutritional status and associated factors among adult TB patients in the Horro Guduru Wollega zone.

## Methods and materials

### Study setting and period

The study was conducted from May 7, 2021, to June 21, 2021, in Horro Guduru Wollega Zone, Oromia Region. The zone is divided into 11 woredas (districts) and one administrative town. Its capital, Shambu town, is 315 kilometres from Ethiopia’s capital, Addis Ababa. The zone has fifty (50) health centres and three (3) hospitals. In each health facility, a TB clinic has been established with at least one TB focal healthcare provider to regularly manage cases and follow their treatment. Fifty (50) health centres and three (3) hospitals in the district provided TB care, including directly observed therapy short course (DOTS), during the study period. During initiation phase anti-TB drug are administrated directly under observation of healthcare provider or TB focal person whereas, in the continuation phase patients receive their anti-TB drugs from the health facilities on every week and manage their pills by themselves.

### Study design and population

A facility-based cross-sectional study design was employed from May 7, 2021, to June 21, 2021. The source population included all adult TB patients taking anti-TB treatment in Horro Guduru Wollega Zone public health centres, while study population included a randomly selected TB patient whose age was ≥ 18 years old taking anti-TB treatment during the study period. TB patients who were 18 years and above, came to TB clinic during the study period were included in the study. However, TB patients who cannot properly communicate, were mentally ill, had physical disabilities, and pregnant or lactating mothers were excluded from the study.

### Sample size determination and sampling procedure

The sample size was calculated using the single population formula (n = Z^α^_2_^2^p (1–p)/d^2^) under the following assumptions: *Z* score at 95% confidence interval CI = 1.96, margin of error = 5 P = 38.9%, the prevalence of under nutrition among TB patients in Shashemane town, central Ethiopia^(15)^ giving a sample size of 365. By considering the design effect of 1.5, it gave 547. Since the number of TB patients who have follow up at a public health centre in the study area was less than 10,000 (N = 798) we used a correction formula which yields a sample size of 324. Adding a 5% no-response rate, the final sample size became 341.

All public health centres in the study area were considered for this study. First, we choose all eleven (11), woredas, and one (1) town administration, by taking into account facilities with a high TB patient flow. Then, two (2) health centres from each woreda were randomly selected. Therefore, a total of 23 health centres were included in the study. Before the beginning the data collection the list of the study participants were obtained from TB registration log book of respective health centres and used as a sampling frame. The required numbers of participants who will be selected from each health centre were determined based on proportion to population size allocation. Finally, study participants were selected using a computer-generated simple random sampling technique from TB registration available in the TB clinic.

### Data collection tools and techniques

**Questionnaires:** The structured questionnaires which are adopted from previous studies.^(14,17,20)^ was employed. Primary data was collected using a structured questionnaire, which included background information, household income, participants’ and/or household food insecurity status, dietary diversity scores, and lifestyle behaviours. Household food security status was assessed using the Household Food Insecurity Access Scale (HFIAS), and dietary diversity scores were assessed using a food frequency questionnaire which was then converted into dietary diversity tertiles by computing for the groups.

**Weight:** Weight was measured using a regularly calibrated beam balance. Clients were instructed to remove shoes and heavy clothing. Participants stood straight and unassisted in the centre of the weighing scale platform. Weight was measured to the nearest 0.1 kg. The validity of the scale was checked by using an object of known weight every morning and between the measurements.

**Height:** Height was measured with a stadiometer, which was readily available and routinely used in the TB clinic. The participants were instructed to take off their shoes and stand erect, looking straight in a vertical plane, feet together, and knees straight. The five contact points (heels, calves, buttocks, shoulder blades, and the back of the head) were designed to make contact with the stadiometer’s wall. The height was measured to the nearest 0.1 cm (centimetre). Before starting the measurement, the stadiometer was checked using calibration rods.^(21)^

All anthropometric measurements were taken in triplicate, and the average results were used in subsequent analyses. Anthropometrics were standardised in order to reduce inter observer error. The body mass index (BMI) was calculated as the weight in kg divided by the height in metres squared (kg/m^2^).

The questionnaire was prepared in English first, then translated into Afan Oromo, and finally back to English to ensure consistency by a bilingual expert. Data were collected by six (6) trained health professionals who had prior knowledge of TB and worked in TB clinics. Two BSc nurses were assigned as supervisors to monitor the data collection process.

The data quality was ensured by carefully designing, translating, and pretesting questionnaires. Before data collection, the questionnaires were pretested on a 5% sample size in an area other than the actual data collection area. Unclear or ambiguous questions were modified, and incorrect skip patterns were corrected. Two days of training were provided for data collectors and supervisors by the principal investigator on data collection tools, data collection techniques, approach to the interviews, and maintaining the privacy and confidentiality of the respondents. Every day following data collection, the questionnaires were reviewed and checked for completeness by the supervisors and the principal investigator. The scale pointer was checked to be zero before taking each measurement.

### Operational definitions

**Severe chronic energy deficiency**: In this study, refers to study participants whose BMI < 15.5 for males and < 20.6 kg/m^2^ for females.

**Mild to moderate chronic energy deficiency**: 15.5–18.2 for males and 20.6–21.8 kg/m^2^ for females.

**Normal nutritional status:** 18.3–21.5 for males and 21.9–23.0 kg/m^2^ for females.

**Overweight:** 21.6–22.2 for males and 23.1–24.5 kg/m^2^ for females

**Obese:** above 22.2 kg/m^2^ for males and **>** 24.5 kg/m^2^ for females.^(22)^

**Nutritional care and support:** It has many components which includes the provision of water, hygiene and food safety service or food aid by any organisation. It was measured by dichotomous yes or no questions, participants who get at least a service in one of the components in the last four weeks were considered as those who have nutrition care and support.^(23)^

**Household food security:** It was measured by using the HFIAS measurement tool, which consists of nine (9) item occurrence questions that represent a generally increasing level of severity of food insecurity (access), and each item has three frequency of occurrence questions asked to determine whether the condition occurred rarely (once or twice), sometimes (three to ten times), or frequently (more than ten times) in the previous four weeks. Each frequency question weighs from 1 to 3 points, giving a total score of 27. Based on the responses given to the nine questions and the frequency of their occurrence over the past 30 d, participants were assigned a score that ranged from 0 to 27. Finally, an index was dichotomised into ‘food-insecure households’ for scores greater than one and ‘food-secure households’ for scores zero and one.^(24–26)^ According to the HFIAS score, there are three categories of food insecurity: mildly food-insecure (if Q1a = 2 or Q1a = 3 or Q2a = 2 or Q2a = 3 or Q3a = 1 or Q4a = 1), moderately food-insecure (if Q3a = 2 or Q3a = 3 or Q4a = 3 or Q5a = 1 or Q5a = 2 or Q6a = 1 or Q6a = 2), severely food-insecure (if Q5a = 3 or Q6a = 3 or Q7a = 1 or Q7a = 2 or Q7a = 3 or Q8a = 1 or Q8a = 2 or Q8a = 3 or Q9a = 1 orQ9a = 2 or Q9a = 3).^(26,27)^

**Food Frequency Questionnaire:** Participants were asked about the usual frequency of consumption of each of the 22 food items they have consumed over the last month last three months. Participants were asked to report the frequency of consumption of each food per day, per week or per month using the past 1 month as a reference. The food frequency questionnaire was calculated using the product sum method. The average frequency of food intake per week and month of the food frequency questionnaire was converted to a daily frequency value. Consumer definitions were done and computed for nine groups of foods. The participants were coded as a ‘consumers’ of a food item if they had consumed the food item at least once per week^(28)^

**Dietary diversity score**: The total count of food groups that an individual consumed based on the DDS guideline of nine food groups was measured by using food frequency questionnaires that consisted of 22 food items. These food items were grouped in to 9 (nine), then their sum product were ranked in to tertiles as low dietary diversity score = <3 food groups, medium dietary diversity = 4 and 5 food groups, and high dietary diversity = > 6 food groups.^(26)^

**Cigarette smokers:** Participants who had been smoking cigarettes for more than 6 months and smoked a minimum of one packet of cigarettes per week.^(29)^

**Problem with eating:** A patient who has at least one of the problems (mouth ulcer, nausea and/ or vomiting, poor appetite, or pain or difficulty of swallowing).

**Intensive phase:** The first two months of period by which anti-TB drugs is given every day under the direct observation of the healthcare provider, with the aim of rapidly reducing the number of mycobacterium tuberculosis present in the patient’s body and minimising the risk of transmission.^(30)^

**Continuation Phase:** The period ranges from 4 to 6 months by which anti-TB drug is given with aim of killing the remaining mycobacterium tuberculosis from the previous stage to prevent recurrence.^(30)^

### Data processing and analysis

Data were visually checked and entered into EpiData version 4.6 and then exported to SPSS version 26 software package (manufactured by IBM®) for analysis. Descriptive analysis was carried out to describe study participants using figures and tables.

The dependent variable was the patient’s nutritional status, which was categorised into normal, undernutrition, and overnutrition based on body mass index. The sociodemographic characteristics, general health and nutrition-related factors, household food security status, and dietary diversity score were the independent variables. A bivariable multinomial logistic regression model was performed to select candidate variables for multinomial logistic regression analysis to identify independent predictors of nutritional status. Variables having a P-value of 0.25 in the bivariable analysis were then included in the multivariable multinomial logistic regression model. Variables with a P-value of < 0.05 in the multivariable multinomial logistic regression analysis were considered independent predictors of nutritional status.

Multicollinearity among independent variables was checked by considering the standard error and tolerance. A risk of multicollinearity among the independent variables was defined as a standard error greater than 2 and a variance inflation factor (VIF) greater than 10. For the finally fitted multivariable logistic regression model, the adequacy of the model to predict the outcome variables was checked by the Hosmer–Lemeshow goodness-of-fit test (P = 0.341).

### Ethical approval

This study was conducted according to the guidelines laid down in the Declaration of Helsinki, and all procedures were approved by the ethical review board of Jimma University’s Institute of Health Science (Ref. No. IHR47/2021). Informed written consent was obtained from all subjects/patients.

## Results

### Sociodemographic characteristics

The study included 334 adult TB patients with a response rate of 97.9%. More than half of the respondents (54.1%) were between the ages of 18 and 35 years. The mean ± SD (standard deviation) age of the respondents was 37 (±13.936) years with a range from 18 to 87 years. About two-thirds of the respondents (66.5%) were from rural areas. Almost two-thirds of the respondents, 201 (60.2%), were married. More than a quarter of the respondents (27.5%) were unable to read and write. Approximately two-thirds (62.3%) of the participants had fewer than or equal to five family members, while the remaining 37.7% had more than five family members. One-third (35.3%) of study participants were farmers by occupation (Table 1).

**Table 1.** Sociodemographic characteristics of adult TB patients in public health centres in Horo Guduru Wollega Zone, Oromia Region, West Ethiopia, 2021

Variable (n = 334)	Category	Number	Percent
Gender	Male	185	55.4
	Female	149	44.6
Educational status	Unable to write and read	92	27.5
	Able to read and write	172	51.5
	College and above	70	21
Age category	18–26	93	27.8
	27–35	88	26.3
	36–44	52	15.6
	45–54	55	16.5
	>=55	46	13.8
Wealth Index	Poor	116	34.7
	Medium	78	23.4
	Rich	140	41.9
Marital status	Single	88	26.3
	Married	201	60.2
	Divorced and widowed	46	13.5
Occupation	Student	64	19.2
	Farmer	118	35.3
	Employee	53	15.9
	Others^a^	99	29.6
Residence	Urban	112	33.5
	Rural	222	66.5
Family size	Less than or equal to 5	208	62.3
	Greater than 5	126	37.7

a Housewife, daily labourer, and merchant.

### General health and nutrition information related characteristics

More than half (58.4%) of the participants had smear-positive pulmonary TB, whereas 25.4% and 16.2% were smear-negative pulmonary tuberculosis (PTB) and extrapulmonary tuberculosis (EPTB), respectively. Regarding their treatment phase, 37.4% were in the intensive phase of anti-TB treatment. On the other hand, 270 (80.8%) and 64 (19.2%) patients were new cases and relapses, respectively.

About half (49.1%) of the participants had problems with eating, whereas 47.6% of these study participants had comorbidities with chronic diseases. About two-thirds (196, 58.7%) of the participants had working functional status. Of the participants, 15.6% had HIV/TB co-infection. About two-thirds, (59.6%) of these study participants received dietary counselling, while 135 (40.4%) didn’t. About one-third (32.0%) of the participants received nutritional care and support. More than half (56.6%) of study participants ate meals less than or equal to three times per day.

In this study, more than one-third, 107 (32.0%), were food-insecure. The food frequency questionnaire was used to generate the dietary diversity score tertiles, more than half (57.5%) of the study participants had a medium dietary diversity, 24.0% of them had a low, and 18.6% of them had a high dietary diversity score (Table 2).

**Table 2.** General health and diet related information of adult TB patients in public health centres of Horro Guduru Wollega Zone, Western Ethiopia, 2021

Variable (n = 334)	Category	Number	Percent
Received dietary counselling	No	135	40.4
	Yes	199	59.6
Having nutritional care and support	No	227	68.0
	Yes	107	32.0
Interruption of treatment	No	209	62.6
	Yes	125	37.4
Reasons for interruptions of the treatment (n = 125)	Side effects of anti-TB drugs	14	4.2
	Sickness/illness	27	8.1
	Lack of enough food to eat	22	6.6
	Boring of taking drugs	23	6.9
	Health institution is far	40	6.3
Having eating problems (e.g. mouth ulcer, vomiting)	No	170	50.9
	Yes	164	49.1
Having comorbidity with other diseases	No	175	52.4
	Yes	159	47.6
TB patient category	New case	270	80.8
	Relapse	64	19.2
Types of TB	PTB smear-positive	195	58.4
	PTB smear-negative	85	25.4
	EPTB	54	16.2
Phase of the treatment	Initiation phase	135	37.4
	Continuation phase	209	62.6
HIV status of the patient	Positive	52	15.6
	Negative	282	84.4
Meal frequency category	Less than 3 times per day	189	56.6
	Greater than or equal to 3	145	43.4
Duration of symptoms before diagnosis	Less than or equals 2weeks	87	26.0
	3–4 Weeks	141	42.2
	Greater than 4 weeks	106	31.7
Functional status	Working	196	58.7
	Ambulatory	138	41.3
HHFS category	Food-secure	227	68.0
	Food-insecure	107	32.0
Dietary diversity score	Low dietary diversity	80	24.0
	Medium dietary diversity	192	57.5
	High dietary diversity	62	18.6

PTB, pulmonary tuberculosis; EPTB, extrapulmonary tuberculosis; HHFS, household food security; DDS, dietary diversity score.

### Nutritional status of adult tuberculosis patients in public health centres of Horro Guduru Wollega Zone, Western Ethiopia

**Figure 1.**
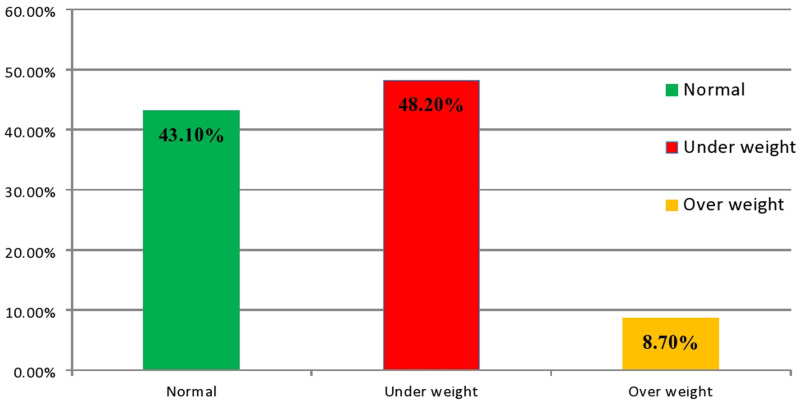
Nutritional status of adults with tuberculosis in public health centres of Horro Guduru Wollega Zone, Western Ethiopia, 2021.

The mean value of BMI of the study participant was 20.14 ± 1.89 SD. The minimum and maximum values were 14.88 and 25.39, respectively. Based on the body mass index (BMI) optimal cut-off points for obesity and markers of metabolic syndrome for Ethiopian adults, 26 (7.8%) of the study participants had severe chronic energy deficiency, 135 (40.4%) had mild to moderate chronic energy deficiency, 144 (43.1%) had normal weight, 19 (5.7%) were overweight, and 10 (3.3%) were obese. Overall, 161 (48.2%) and 29 (8.7%) of the participants were categorised as having undernutrition and overnutrition, respectively (Fig. 1).

### Factors associated with nutritional status of adult tuberculosis patients

In the bivariable multinomial logistic regression, gender, family size, drinking alcohol, treatment phase, household food security status, dietary diversity score, eating problem, nutritional care and support, dietary counselling, and meal frequency had a P-value <0.25 and were used to adjust the odds ratio in the multinomial logistic regression analysis.

In the multivariable multinomial logistic regression analysis, gender, treatment phase, dietary counselling, and meal frequency were found to be independent predictors of nutritional status in adult TB patients.

Accordingly, in the multivariable logistic regression analysis, female TB patients (AOR = 3.48, 95% CI: (1.918–6.314)), TB patients who had no dietary counselling (AOR = 2.51, 95% CI: (1.335–4.720)), TB patients in the initiation phase of the treatment (AOR = 3.77, 95% CI: (2.072–6.852)), and TB patients who ate less than three times per day (AOR = 3.6, 95% CI: (1.942–6.676)) were factors significantly associated with under nutrition (Table 3).

**Table 3.** Multivariable multinomial logistic regression analysis showing factors associated with nutritional status of the adults with tuberculosis in public health centres of Horro Guduru Wollega Zone, Western Ethiopia, 2021

Variables	Category	Nutritional status			Undernutrition			Over nutrition		
		Normal weight (%)	Undernutition (%)	Overnutrition (%)	COR (95% CI)		AOR (95% CI)	COR (95% CI)		AOR (95% CI)
Sex	Female	41 (12.3)	96 (28.7)	12 (3.6)	0.54 (0.40–0.73)		**3.48 (1.92–6.31)** ^*^	0.79 (0.59–1.06)		2.09 (0.82–5.35)
	Male	103 (30.8)	65 (19.5)	17 (5.1)	1			1		
Family size	Greater than 5	48 (14.4)	97 (19.2)	15 (4.2)	0.85 (0.65–1.11)		1.36 (0.73–2.53)	1.20 (0.92–1.57)		2.13 (0.78–5.76)
	Less than or equals 5	96 (28.7)	64 (29.0)	14 (4.5)	1			1		
Drinking alcohol	Yes	49 (14.7)	70 (21.0)	10 (3.0)	0.80 (0.61–1.05)		1.21 (0.65–2.26)	1.14 (0.87–1.50)		0.876 (0.32–2.41)
	No	95 (28.4)	91 (27.2)	19 (5.7)	1			1		
Treatment phase	Initiation Phase	29 (8.7)	73 (26.3)	8 (2.4)	0.55 (0.414–0.72)		**3.76 (2.07–6.85)** ^*^	0.81 (0.61–1.06)		1.250 (0.45–3.44)
	Continuation Phase	115 (34.4)	88 (21.9)	21 (6.3)	1			1		
Food Security Status	Food-secure	106 (31.7)	98 (29.3)	23 (6.9)	1			1		
	Food-insecure	38 (11.4)	63 (18.9)	6 (1.8)	0.78 (0.604–1.010)		0.862 (0.45–1.66)	1.11 (0.86–1.44)		0.585 (0.18–1.84)
Dietary diversity score	Low DDS	32 (9.6)	43 (12.9)	5 (1.5)	0.573 (0.354–0.929)		1.084 (0.43–2.74)	0.81 (0.50–1.32)		0.91 (0.22–3.78)
	Medium DDS	79 (23.7)	97 (29.0)	16 (4.8)	0.51 (0.27–0.98)		1.64 (0.75–3.54)	0.56 (0.33–0.98)		0.94 (0.32–2.74)
	High DDS	33 (9.9)	21 (6.3)	8 (2.4)	1			1		
					
Eating Problem	Yes	62 (18.6)	87 (26.0)	15 (4.5)	0.762 (0.566–1.026)		0.97 (0.54–1.76)	1.08 (0.80–1.45)		1.59 (0.64–3.95)
	No	82 (24.6)	74 (22.2)	14 (4.2)	1			1		
Nutritional care and support	No	89 (26.6)	120 (35.9)	18 (5.4)	0.645 (0.445–0.935)		0.85 (0.44–1.63)	0.92 (0.64–1.33)		0.757 (0.27–2.08)
	Yes	55 (16.5)	41 (12.3)	11 (3.3)	1			1		
Dietary counselling	No	35 (10.5)	89 (26.6)	11 (3.3)	0.56 (0.426–0.750)		**2.510 (1.33–4.72)** ^*^	0.827 (0.63–1.09)		1.774 (0.65–4.87)
	Yes	109 (32.6)	72 (21.6)	18 (5.4)	1			1		
					
Meal frequency	Less than 3	56 (16.8)	121 (36.2)	12 (3.6)	0.42 (0.29–0.58)		**3.60 (1.94–6.67)** ^*^	0.62 (0.45–0.86)		1.145 (0.42–3.09)
	Greater or equals 3	88 (26.3)	40 (12.0)	17 (5.1)	1			1		
HIV status	Positive	14 (4.2)	31 (9.3)	7 (2.1)	0.84 (0.66–1.06)		1.550 (0.68–3.508)	1.194 (0.94–1.50)		2.517 (0.791–8.01)
	Negative	130 (38.9)	130 (38.9)	22 (6.6)	1			1		

* Statistically significant variables in multivariable logistic regression at P-value <0.05, COR, Crude Odds Ratio; AOR, Adjusted Odds Ratio.

## Discussion

According to this study, 48.2% of TB patients in the study area were undernourished. Sex, duration of treatment, dietary counselling, and meal frequency were all factors significantly associated with undernutrition among adult TB patients.

The prevalence of undernutrition in this study is higher compared to studies conducted in Nepal (36.1%), Sri Lanka (37.8%), Burkina Faso (35.8%),^(31–33)^ Shashemane town (38.9%),Addis Ababa (28.5%), Hossaina town (39.7%), Somali Region Jijiga town (44.3%), and Dire Dawa town (37.9%),^(18,23,34–36)^ while it is lower compared to studies conducted in India (79.5%) and in different parts of Ethiopia such as Bale zone (63.2%), Adama town (53%), Amhara Region (57.17%), and Gondar town (71.35%).^(15,21,36–38)^ The inconsistency could be attributed to differences in socio-economic status across the study population and study period.

In this study, the prevalence of over nutrition was 8.7%, and no variable was statistically significant for this status. Moreover, this finding was lower compared to studies conducted in Nepal (11.35%,^(32)^ Sri Lanka (9.7%,^(33)^ and India (9.3%),^(24)^ and higher than a study conducted in Burkina Faso (2.6%)^(31)^ and Kenya (2.0%),^(39)^ Southern India (14.7%).^(40)^The difference may be explained by differences in socio-economic status and the tools used; in this study, BMI cut-off points for Ethiopian adults were used to classify the nutritional status.

Female TB patients were about 3.5 times more likely to be undernourished when compared to male patients. This finding is consistent with other studies done in Adama, Jijiga Town, Amhara Region, Ethiopia.^(18,36,38)^ This could be because women in Africa, including Ethiopia, have fewer educational and employment opportunities, resulting in limited access to healthcare and nutritious food. Females and children have lower priority in the family for nutritious food than males. In addition, women’s diets are influenced by various factors, especially food access and affordability, gender inequality, and social and cultural norms may constrain women’s ability to make decisions about their nutrition and care.^(41)^

In this study, patients who did not receive dietary counselling were 2.5 times more likely to be undernourished compared with those who received it. This is consistent with a study conducted in Addis Ababa.^(23)^ This could be because TB patients who have received dietary counselling are more aware of dietary issues. As a result, they may consume an adequate quantity and quality of a variety of foods, preventing malnutrition. This finding is more supported by study conducted in Ludhiana city hospitals in India by which nutritional counselling brought favourable and significant changes in the nutritional profile of patients with chronic diseases. It has been considered as a component in the management of malnutrition among patients with chronic disease.^(42)^

Participants who were in the initiation phase of treatment had 3.7 times higher chance of being undernourished compared to those patients in the continuation phase. This was consistent with the study done in India.^(43)^ This could be explained by the fact that patients who took anti-TB drugs for up to two months expected to begin to recover from the illness when they experienced fewer episodes of vomiting, nausea, and loss of appetite, which led to an improvement in their nutritional profile.

Study participants who ate less than three times per day were 3.6 times more likely to have undernutrition than those who ate greater than or equal to three times per day. This finding supports previous research from Burkina Faso,^(31)^ and Gondar, Northern Ethiopia.^(37)^ This mi ght be attributed to more frequent meal intake per day, which will correct nutritional status and enhance the impact of TB treatment by supporting the body’s immune system and promoting weight gain. This finding is more supported by the study conducted in India, where an increase in eating frequency was correlated with an increase in the prevalence of normal BMI among the study participants.^(44)^

### Limitation

The main limitation of the study is the lack of information on the duration of the disease and nutritional status before the onset of the disease since the study was conducted on patients taking anti-TB drugs regardless of their previous nutritional status before the onset of the disease. And also the portion size of each food item was not included in the food frequency questionnaire. Lack of other markers for nutritional status such as vitamin D, A or hemoglobin values was also another limitation of this study. In this study, there were small numbers of patients with overnutrition status which may have limited power to detect associations. Data collectors were trained on some techniques of reducing bias introduced due to a verbal report of the study participants.

## Conclusion and recommendation

In this study, the magnitude of undernutrition among adult TB patients was high, showing disparities and slight similarities with studies conducted in some parts of Ethiopia. Being a female, being on the initiation phase of treatment, lack of dietary counselling, and having meal less than three per day were independently associated with under nutrition. However, no variables were found to be statistically significant in over nutrition TB patients. As a result, a regular nutritional assessment and dietary counselling for all TB patients should be part of routine care, particularly for female patients, and patients in the intensive phase of treatment should be encouraged at all facilities that provide TB clinic services. A food safety net should be established for those who eat fewer than three times per day.

## Data Availability

The data sets analysed during the current study are available from the corresponding author on reasonable request.
